# Corticosteroids combined with infliximab vs. corticosteroids sequential infliximab for acute severe ulcerative colitis with mucosal deficiency: a retrospective study

**DOI:** 10.3389/fmed.2024.1442519

**Published:** 2024-11-20

**Authors:** Xiaolei Liu, Xianmin Xue, Xiaojing Zhu, Jun Liu, Yongquan Shi, Min Chen

**Affiliations:** ^1^Department of Medical Insurance, Xijing Hospital, Air Force Military Medical University, Xi’an, China; ^2^State Key Laboratory of Cancer Biology, National Clinical Research Center for Digestive Diseases and Xijing Hospital of Digestive Diseases, Air Force Military Medical University, Xi’an, China

**Keywords:** corticosteroids, infliximab, acute severe ulcerative colitis, mucosal deficiency, combined, sequential

## Abstract

**Introduction:**

Mucosal deficiency is one of the most challenging conditions in patients with acute severe ulcerative colitis (ASUC). Intravenous corticosteroids (CS) are the first-line treatment, with infliximab (IFX) used as a rescue therapy. However, the efficacy remains unsatisfactory. We investigated whether CS combined with IFX as first-line therapy would improve outcomes in patients with ASUC with mucosal deficiency.

**Methods:**

A retrospective study was performed at a tertiary inflammatory bowel disease center. The primary outcomes included clinical remission, endoscopic improvement, and endoscopic remission at week 14. The secondary outcomes included the colectomy rate within 90 days and durable clinical remission.

**Results:**

A total of 43 patients with ASUC with mucosal deficiency were included in the analysis (25 in the CS combined with the IFX group and 18 in the CS sequential IFX group). At week 14, endoscopic improvement was observed in 21 of 25 patients (84.0%) receiving the CS combined with the IFX regimen, compared to 9 of 18 (50.0%) patients receiving the CS sequential IFX regimen (*p* = 0.017). Durable clinical remission rates were significantly higher in the combined group than in the sequential group (85.7% vs. 35.7%, *p* = 0.004). There was no statistically significant difference between the two groups in terms of clinical and endoscopic remission at week 14 or colectomy rate within 90 days. Multivariate analysis confirmed that the CS combined with the IFX regimen was an independent predictive factor for a higher endoscopic improvement rate at week 14 (odds ratio (OR) 8.428, 95%confidence interval (CI) 1.539–46.153, *p* = 0.014) and a higher durable clinical remission rate (OR 10.800, 95%CI 2.095–55.666, *p* = 0.004).

**Conclusion:**

CS combined with IFX as first-line therapy may be an effective induction strategy in patients with ASUC with mucosal deficiency. Further large-scale, multicenter prospective studies are needed.

## Introduction

Acute severe ulcerative colitis (ASUC) is a life-threatening condition (1). Approximately 25% of patients with ulcerative colitis (UC) will require hospitalization due to an episode of ASUC (2). ASUC is generally diagnosed according to the Truelove and Witts criteria, which include a bloody stool frequency of ≥6 per day and at least one of the following: pulse rate > 90 bpm, temperature > 37.8°C, hemoglobin <10.5 g/dL, and erythrocyte sedimentation rate > 30 mm/h (3). For decades, intravenous corticosteroids (CS) with rapid induction have been the standard first-line medical treatment for ASUC. However, despite its effectiveness, up to 30–40% of patients do not respond to this treatment alone and require second-line therapy (4, 5). Previous studies have demonstrated that infliximab (IFX) and ciclosporin are effective second-line rescue therapies (6). Meanwhile, various rescue strategies have been explored, including accelerated induction with IFX therapy (7) and small-molecule Janus kinase (JAK) inhibitors such as tofacitinib or upadacitinib (8–10). Surprisingly, despite therapeutic advances, there still exists a significant rate of treatment failure, which typically results in colectomy. This highlights the need for more effective therapies for patients with ASUC.

In clinical practice, mucosal deficiency is one of the most challenging conditions in patients with ASUC. The patients face the risk of imminent life-threatening deterioration and may require a colectomy. They require a more effective strategy to prevent disease exacerbation in the short term. However, few studies have been conducted on this type of ASUC, which is further diagnosed by colonoscopy. In the clinic, we found that the initial treatment of IFX combined with intravenous CS may be an effective strategy for patients with ASUC with mucosal deficiency.

In this study, we aimed to present a retrospective analysis of patients with ASUC with mucosal deficiency and to compare the efficacy of CS combined with IFX to CS sequential IFX.

## Methods

### Study design and patient selection

**Figure 1:**
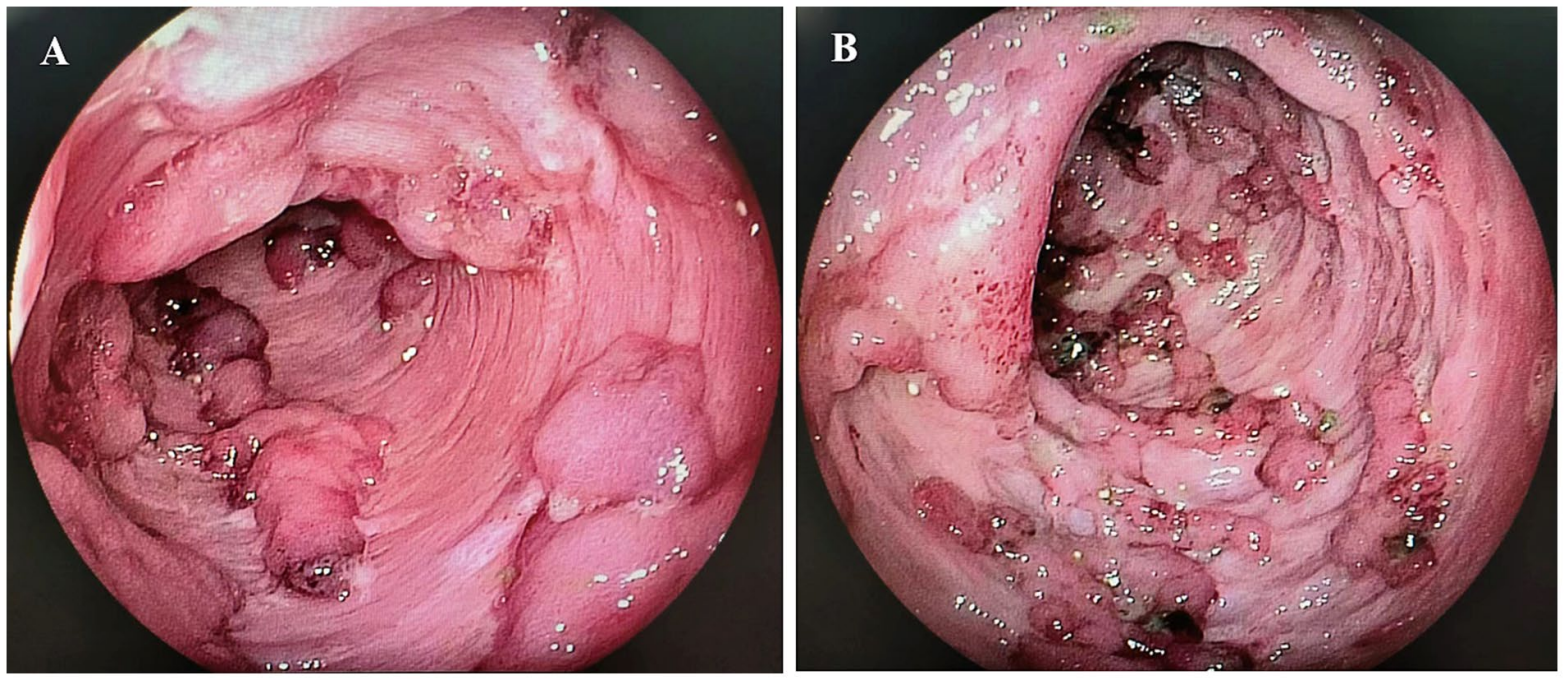
Mucosal deficiency of the colon. (A) Mucosal deficiency in the transverse colon; (B) Mucosal deficiency in the sigmoid colon.

We conducted a retrospective chart review of patients hospitalized with ASUC between January 2019 and April 2023 at a single tertiary IBD center. ASUC was diagnosed according to the Truelove and Witts criteria (3). Patients with ASUC with mucosal deficiency (Figure 1), which was defined as the absence of the colonic mucosa layer, exposure of the muscular layer, and a total length of mucosal deficiency ≥10 cm, were included in the analysis. Patients with a follow-up duration of less than 14 weeks or a history of previous colectomy were excluded. The study was approved by the institutional research ethics committee of Xijing Hospital, the Air Force Military Medical University, and was conducted in accordance with the Declaration of Helsinki.

**Figure 2:**
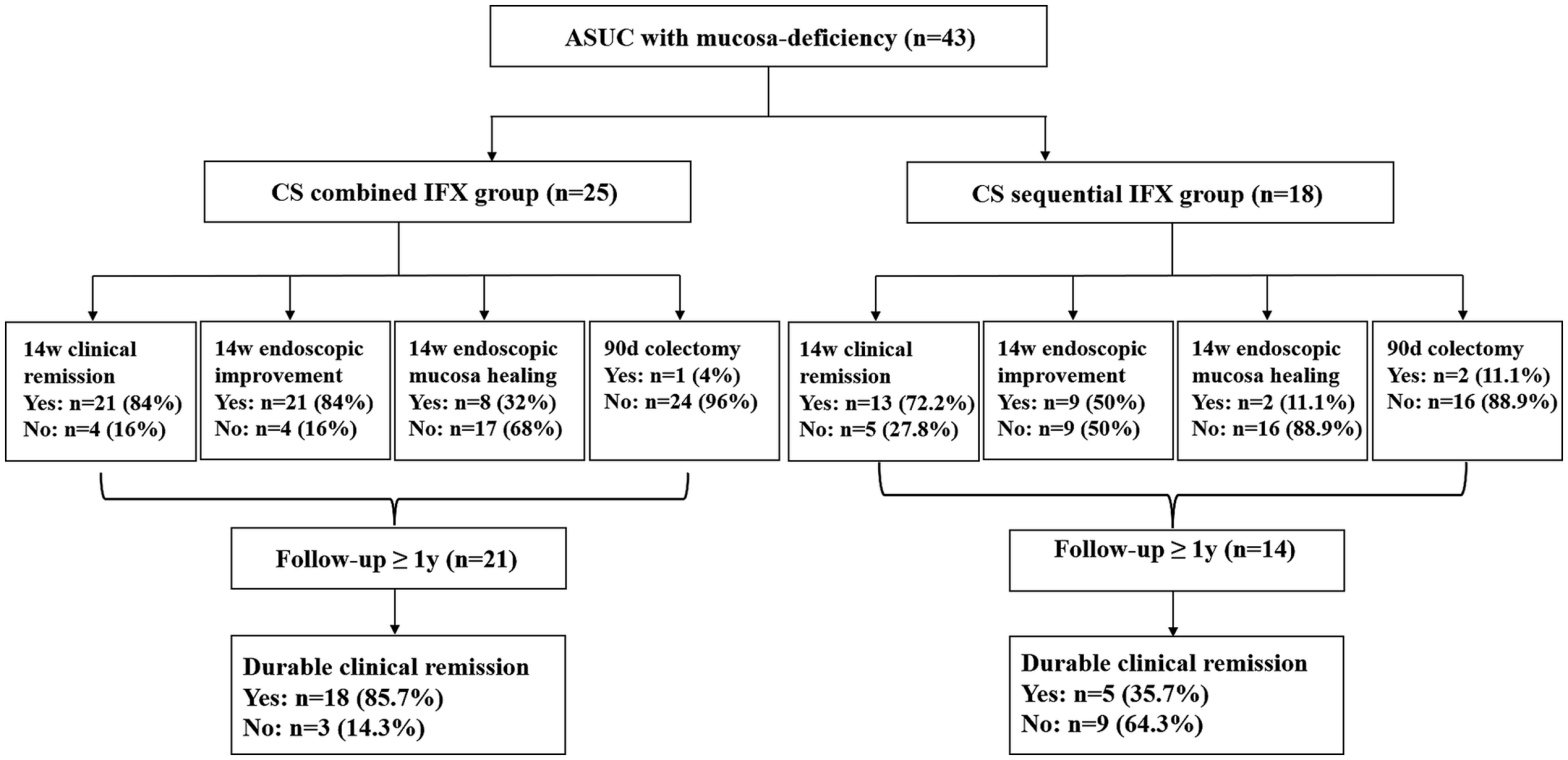
Flowchart of the study groups and patient results. ASUC, acute severe ulcerative colitis; CS, corticosteroids; IFX, infliximab.

Finally, 243 patients with ASUC were identified, 43 of whom were eligible for inclusion and were assigned to two different treatment groups. One group (*n* = 25) had an initial CS combined with the IFX regimen (combined group), meaning that the patients were treated with both CS and IFX simultaneously. The other group (*n* = 18) was treated with a CS sequential IFX regimen (sequential group), meaning that rescue therapy with IFX was initiated 3–5 days after the commencement of CS therapy (Figure 2). The patients received CS with a loading dose of intravenous hydrocortisone (300 mg/day) for 3–5 days, followed by oral prednisone (initial dose 0.75–1 mg/Kg, tapered by 5 mg/week). IFX was administered according to the standard dosing schedule of 5 mg/Kg at weeks 0, 2, and 6. The patients continued to receive maintenance doses every 8 weeks.

### Data collection

The baseline demographic, endoscopic, and clinical information of all patients were collected. The following variables were compared between the two treatment groups: age, sex, body mass index (BMI), disease duration, disease location, prior medication use (oral mesalamine, systematic steroids, immunosuppressants such as azathioprine and methotrexate, and biologics such as IFX, adalimumab, and vedolizumab), extraintestinal manifestations, intestine numbers with mucosal deficiency, Mayo Clinic score, accompanying symptoms (fever and abdominal tenderness), laboratory values (serum albumin, hemoglobin, C-reactive protein [CRP], and erythrocyte sedimentation rate [ESR]), and opportunistic infection tests (*C. difficile*, cytomegalovirus [CMV], and Epstein–Barr virus [EBV]).

Positive CMV or EBV infection was defined as a positive IgM or polymerase chain reaction (PCR) result in the serology test or positive immunohistochemical staining on biopsy. Disease location was evaluated based on the Montreal classification (11). Intestine numbers with mucosal deficiency refer to the number of colon segments involved with mucosal deficiency.

### Outcomes

The primary outcomes were clinical remission (defined as a Mayo Clinic score ≤ 2, with no subscore>1), endoscopic improvement (a decrease in the Mayo endoscopic subscore determined by the physician performing the procedure), and endoscopic remission (a Mayo endoscopic subscore of 0 or 1) at week 14. The secondary outcomes included the colectomy rate within 90 days and durable clinical remission (remission at both weeks 14 and 48).

### Statistical analysis

The continuous variables were presented as mean and standard deviation (SD) and compared with Student’s *t*-tests. The categorical variables were represented as numbers with percentages and compared using Fisher’s exact test or Pearson’s chi-squared test. To estimate odds ratios (ORs) and a 95% confidence interval (95%CI), binary logistic regression models were used. Only the variables with a *p*-value <0.10 in the univariate analysis were included in the multivariate analysis. Statistical significance was considered when the two-sided p-value was <0.05. The Statistical Package for the Social Sciences (SPSS 26.0 Package Facility; SPSS Inc., Chicago, IL, United States) software was used for data analysis.

## Results

### Clinical characteristics of the patients

In total, 43 patients with ASUC with mucosal deficiency were included in the analysis. The median follow-up period was 1.33 years (range: 0.3–4.3 years). A total of 25 patients treated with the CS combined with the IFX regimen and 18 patients treated with the CS sequential IFX regimen were compared. There were 21 patients in the combined group and 14 patients in the sequential group with a follow-up of at least 1 year (Figure 2). The patient demographics are shown in Table 1. Clinical and laboratory parameters were similar in both groups (*p* > 0.05).

**Table 1:** Clinical characteristics of the patients in different treatment groups.

	Combined group (*n* = 25)	Sequential group (*n* = 18)	*p*-value
Age (y, mean ± SD)	43.76 ± 12.33	42.44 ± 14.35	0.75
Sex (female), *n* (%)	11 (44.0)	5 (27.8)	0.28
BMI (Kg/m^2^, mean ± SD)	20.96 ± 3.43	20.54 ± 2.66	0.67
Disease duration (y, mean ± SD)	2.08 ± 3.32	2.69 ± 4.64	0.61
Prior exposure to medication, *n* (%)			
Oral mesalamine	24 (96.0)	18 (100.0)	1.00
Systematic steroids	16 (64.0)	11 (61.1)	0.85
Immunosuppressant	2 (8.0)	2 (11.1)	1.00
Biologics	5 (20.0)	4 (22.2)	1.00
Extraintestinal manifestations, *n* (%)	5 (20%)	4 (22.2%)	1.00
Disease location, *n* (%)			1.00
E1	0 (0%)	0 (0%)	
E2	2 (8%)	2 (11.1%)	
E3	23 (92%)	16 (88.9%)	
Intestine numbers with mucosal deficiency (mean ± SD)	2.36 ± 1.52	2.33 ± 1.08	0.95
Mayo Clinic score (mean ± SD)	11.16 ± 0.37	11.22 ± 0.43	0.62
Fever, *n* (%)	16 (64.0)	8 (44.4)	0.20
Abdominal tenderness, *n* (%)	1 (4.0)	1 (5.5)	1.00
Albumin (g/L, mean ± SD)	29.06 ± 5.12	30.27 ± 5.64	0.48
Hemoglobin (g/L, mean ± SD)	93.96 ± 27.22	99.44 ± 20.81	0.48
CRP (mg/L, mean ± SD)	87.41 ± 65.70	53.37 ± 53.97	0.08
ESR (mm/h, mean ± SD)	54.39 ± 35.49	49.94 ± 27.73	0.67
*C. difficile* infection, *n* (%)	8 (32.0)	4 (22.2)	0.73
CMV infection, *n* (%)	11 (44.0)	10 (55.6)	0.35
EBV infection, *n* (%)	3 (12.0)	4 (22.2)	0.41

SD, standard deviation; BMI, body mass index; E1, proctitis; E2, left-sided colitis; E3, extensive colitis; CRP, C-reactive protein; ESR, erythrocyte sedimentation rate; CMV, cytomegalovirus; and EBV, Epstein–Barr virus.

The majority of the patients had pancolitis (92% in the combined group vs. 88.9% in the sequential group). All patients had severe inflammation, with an average CRP of 87.41 mg/L in the combined group and 53.37 mg/L in the sequential group. Patients also had significant hypoalbuminemia, with an average albumin level of 29.06 g/L in the combined group and 30.27 g/L in the sequential group. Approximately one-fifth of the patients had extraintestinal manifestations, such as arthritis, erythema nodosum, conjunctivitis, and oral ulcers (20% of the combined group vs. 22.2% of the sequential group). Almost one-fifth of the patients were previously treated with biologics (20% of the combined group vs. 22.2% of the sequential group), and two-thirds had been treated with systematic steroids (64% of the combined group vs. 61.1% of the sequential group). The majority of the patients (28/43, 65.1%) had simultaneous involvement of more than two intestinal segments with mucosal deficiency.

### Primary and secondary outcomes

In total, 21 (84.0%) patients in the combined group and 13 (72.2%) patients in the sequential group achieved clinical remission at week 14. However, no difference was observed between the two groups (*p* = 0.455). The endoscopic improvement rate at week 14 was significantly higher in the combined group compared to the sequential group (84.0% vs. 50.0%, *p* = 0.017). The endoscopic remission rates at week 14 were 32.0% (8/25) in the combined group and 11.1% (2/18) in the sequential group; however, there was no difference between the groups (*p* = 0.153).

**Figure 3:**
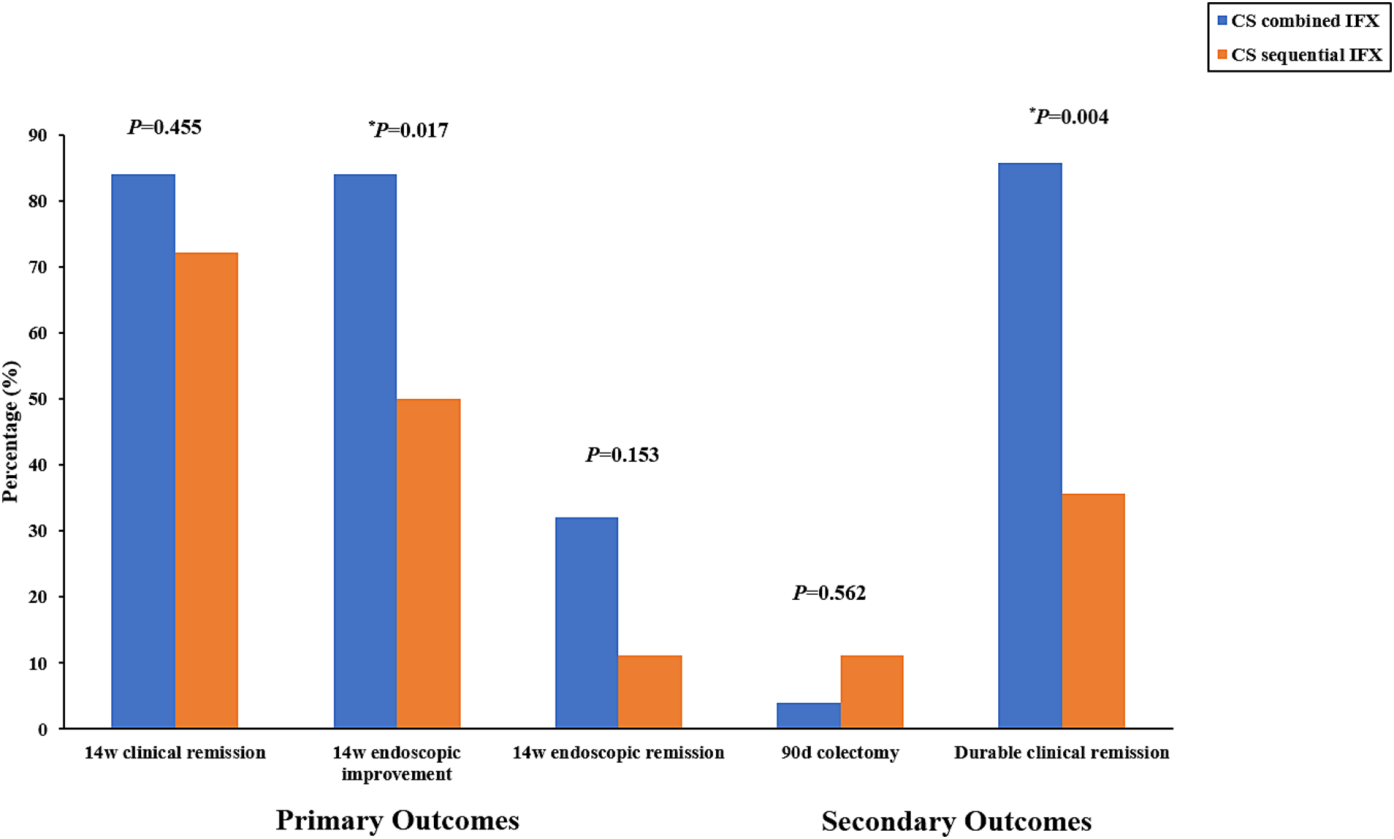
Primary outcomes (rate of clinical remission, endoscopic improvement, and endoscopic remission at week 14) and secondary outcomes (percentage of patients requiring colectomy within 90 days and rate of durable clinical remission). CS, corticosteroids; IFX, infliximab.

For the secondary outcomes, the need for colectomy within 90 days occurred in 1 of 25 (4.0%) patients in the combined group compared to 2 of 18 (11.1%) patients in the sequential group. However, no significant difference was observed between the two groups (*p* = 0.562). In contrast, the durable clinical remission rates were significantly higher in the combined group compared to the sequential group (85.7% vs. 35.7%, *p* = 0.004). The primary and secondary outcomes are shown in Table 2 and Figure 3.

**Table 2:** Clinical outcomes in the different treatment groups.

	Combined group (*n* = 25)	Sequential group (*n* = 18)	*P*-value
14-week clinical remission, *n* (%)	21 (84.0)	13 (72.2)	0.455
14-week endoscopic improvement, *n* (%)	21 (84.0)	9 (50.0)	0.017
14-week endoscopic remission, *n* (%)	8 (32.0)	2 (11.1)	0.153
90-day colectomy, *n* (%)	1 (4.0)	2 (11.1)	0.562
Durable clinical remission, *n* (%)	18/21 (85.7)	5/14 (35.7)	0.004

No serious adverse events directly attributable to the regimens occurred in any of the patients.

### The impact of the different treatments on the primary and secondary outcomes

To compare the impact of the different treatments on the clinical and endoscopic outcomes, univariate and multivariate analyses were performed. The univariate analysis showed that the CS combined with the IFX regimen may be associated with higher rates of endoscopic improvement at week 14 and durable clinical remission. Furthermore, the multivariate analysis revealed similar results, suggesting that the CS combined with the IFX regimen was an independent predictive factor for a higher endoscopic improvement rate at week 14 (OR 8.428, 95%CI 1.539–46.153, *p* = 0.014) and a higher durable clinical remission rate (OR 10.800, 95%CI 2.095–55.666, *p* = 0.004). Tables 3, 4 show the associated results. However, the different treatments were not associated with the rates of clinical remission and endoscopic remission at week 14 and the colectomy rate within 90 days (Supplementary Tables S1–S3).

**Table 3:** Univariate and multivariate analyses of the risk factors of 14-week endoscopic improvement in the participants according to the analyzed variables.

	14w endoscopic improvement	Univariate analysis			Multivariate analysis		
	*n* = 30	OR	95% CI	*P*-value	OR	95% CI	*P*-value
Age (y), *n* (%)							
≤40 (*n* = 19)	14 (73.7)	1					
>40 (*n* = 24)	16 (66.7)	0.714	0.189–2.695	0.619			
Sex, *n* (%)							
Male (*n* = 27)	18 (66.7)	1					
Female (*n* = 16)	12 (75.0)	1.500	0.375–5.988	0.566			
BMI (Kg/m^2^), *n* (%)							
≤20 (*n* = 16)	10 (62.5)	1					
>20 (*n* = 27)	20 (74.1)	1.714	0.454–6.473	0.427			
Disease duration (y), *n* (%)							
<1 (*n* = 19)	14 (73.7)	1					
≥1 (*n* = 24)	16 (66.7)	0.714	0.189–2.695	0.619			
Disease location, *n* (%)							
E2 (*n* = 4)	2 (50.0)	1					
E3 (*n* = 39)	28 (71.8)	2.545	0.318–20.382	0.379			
Mayo clinic score, *n* (%)							
11 (*n* = 35)	25 (71.4)	1					
12 (*n* = 8)	5 (62.5)	0.667	0.133–3.329	0.621			
Extraintestinal manifestations, *n* (%)							
No (*n* = 34)	24 (70.6)	1					
Yes (*n* = 9)	6 (66.7)	0.833	0.173–4.006	0.820			
Prior oral mesalamine use, *n* (%)							
No (*n* = 1)	1 (100.0)	1					
Yes (*n* = 42)	29 (69.0)	0.000	-	1.000			
Prior systematic steroid use, *n* (%)							
No (*n* = 16)	9 (56.3)	1					
Yes (*n* = 27)	21 (77.8)	2.722	0.712–10.409	0.143			
Prior azathioprine use, *n* (%)							
No (*n* = 39)	27 (69.2)	1					
Yes (*n* = 4)	3 (75.0)	1.333	0.126–14.165	0.811			
Prior biologics use, *n* (%)							
No (*n* = 34)	25 (73.5)	1					
Yes (*n* = 9)	5 (55.6)	0.450	0.098–2.057	0.303			
Fever, *n* (%)							
No (*n* = 19)	11 (57.9)	1					
Yes (*n* = 24)	19 (79.2)	2.764	0.722–10.571	0.138			
Abdominal tenderness, *n* (%)							
No (*n* = 41)	28 (68.3)	1					
Yes (*n* = 2)	2 (100.0)	-	-	0.999			
CRP at induction (mg/L), *n* (%)							
≤40 (*n* = 18)	11 (61.1)	1					
>40 (*n* = 25)	19 (76.0)	2.015	0.539–7.538	0.298			
Albumin at induction (g/L), *n* (%)							
≤25 (*n* = 9)	4 (44.4)	1					
>25 (*n* = 34)	26 (76.5)	4.062	0.875–18.858	0.073	7.685	1.147–51.505	0.036
CRP/albumin ratio, *n* (%)							
≤1.7 (*n* = 22)	15 (68.2)	1					
>1.7 (*n* = 21)	15 (71.4)	1.167	0.317–4.299	0.817			
*C. difficile* infection, *n* (%)							
No (*n* = 31)	22 (71.0)	1					
Yes (*n* = 12)	8 (66.7)	0.818	0.196–3.415	0.783			
CMV infection *n* (%)							
No (*n* = 22)	14 (63.6)	1					
Yes (*n* = 21)	16 (76.2)	1.829	0.485–6.898	0.373			
EBV infection, *n* (%)							
No (*n* = 36)	26 (72.2)	1					
Yes (*n* = 7)	4 (57.1)	0.513	0.097–2.711	0.432			
Therapy, *n* (%)							
CS sequential IFX (*n* = 18)	9 (50.0)	1					
CS combined with IFX (*n* = 25)	21 (84.0)	5.250	1.278–21.571	0.021	8.428	1.539–46.153	0.014

BMI, body mass index; E2, left-sided colitis; E3, extensive colitis; CRP, C-reactive protein; CMV, cytomegalovirus; EBV, Epstein–Barr virus; CS, corticosteroids; and IFX, infliximab.

**Table 4:** Univariate and multivariate analyses of the risk factors of durable clinical remission in the participants according to the analyzed variables.

	Durable clinical remission	Univariate analysis			Multivariate analysis		
	*n* = 23	OR	95% CI	*P*-value	OR	95% CI	*P*-value
Age (y), *n* (%)							
≤40 (*n* = 18)	12 (66.7)	1					
>40 (*n* = 17)	11 (647)	0.917	0.227–3.704	0.903			
Sex, *n* (%)							
Male (*n* = 21)	12 (57.1)	1					
Female (*n* = 14)	11 (78.6)	2.750	0.589–12.849	0.198			
BMI (Kg/m^2^), *n* (%)							
≤20 (*n* = 14)	9 (64.3)	1					
>20 (*n* = 21)	14 (66.7)	1.111	0.268–4.600	0.884			
Disease duration (y), *n* (%)							
<1 (*n* = 13)	10 (76.9)	1					
≥1 (*n* = 22)	13 (59.1)	0.433	0.092–2.031	0.289			
Disease location, *n* (%)							
E2 (*n* = 4)	2 (50.0)	1					
E3 (*n* = 31)	21 (67.7)	2.100	0.257–17.143	0.489			
Mayo clinic score, *n* (%)							
11 (*n* = 30)	21 (70.0)	1					
12 (*n* = 5)	2 (40.0)	0.286	0.041–2.013	0.208			
Extraintestinal manifestations, *n* (%)							
No (*n* = 30)	19 (63.3)	1					
Yes (*n* = 5)	4 (80.0)	2.316	0.229–23.417	0.477			
Prior oral mesalamine use, *n* (%)							
No (*n* = 1)	1 (100.0)	1					
Yes (*n* = 34)	22 (64.7)	0.000	-	1.000			
Prior systematic steroid use, *n* (%)							
No (*n* = 11)	6 (54.5)	1					
Yes (*n* = 24)	17 (70.8)	2.024	0.462–8.869	0.350			
Prior azathioprine use, *n* (%)							
No (*n* = 31)	20 (64.5)	1					
Yes (*n* = 4)	3 (75.0)	1.650	0.153–17.824	0.680			
Prior biologics use, *n* (%)							
No (*n* = 28)	19 (67.9)	1					
Yes (*n* = 7)	4 (57.1)	0.632	0.116–3.437	0.595			
Fever, *n* (%)							
No (*n* = 15)	8 (53.3)	1					
Yes (*n* = 20)	15 (75.0)	2.625	0.626–11.002	0.187			
Abdominal tenderness, *n* (%)							
No (*n* = 33)	21 (63.6)	1					
Yes (*n* = 2)	2 (100.0)	-	-	0.999			
CRP at induction (mg/L), *n* (%)							
≤40 (*n* = 15)	9 (60.0)	1					
>40 (*n* = 20)	14 (70.0)	1.556	0.381–6.357	0.538			
Albumin at induction (g/L), *n* (%)							
≤25 (*n* = 5)	2 (40.0)	1					
>25 (*n* = 30)	21 (70.0)	3.500	0.497–24.654	0.208			
CRP/albumin ratio, *n* (%)							
≤1.7 (*n* = 19)	13 (68.4)	1					
>1.7 (*n* = 16)	10 (62.5)	0.769	0.190–3.120	0.713			
*C. difficile* infection, *n* (%)							
No (*n* = 27)	17 (63.0)	1					
Yes (*n* = 8)	6 (75.0)	1.765	0.297–10.472	0.532			
CMV infection *n* (%)							
No (*n* = 18)	11 (61.1)	1					
Yes (*n* = 17)	12 (70.6)	1.527	0.737–6.252	0.556			
EBV infection, *n* (%)							
No (*n* = 30)	19 (63.3)	1					
Yes (*n* = 5)	4 (80.0)	2.316	0.229–23.417	0.477			
Therapy, *n* (%)							
CS sequential IFX (*n* = 14)	5 (35.7)	1					
CS combined with IFX (*n* = 21)	18 (85.7)	10.800	2.095–55.666	0.004	10.800	2.095–55.666	0.004

BMI, body mass index; E2, left-sided colitis; E3, extensive colitis; CRP, C-reactive protein; CMV, cytomegalovirus; EBV, Epstein–Barr virus; CS, corticosteroids; and IFX, infliximab.

### The impact of other potential factors on the primary and secondary outcomes

The multivariate analysis showed that a serum albumin level greater than 25 g/L at induction was associated with higher rates of endoscopic improvement at week 14 (OR 7.685, 95%CI 1.147–51.505, *p* = 0.036) and clinical remission at week 14 (OR 10.625, 95%CI 1.258–89.737, *p* = 0.030). Although age, fever, and CRP levels at induction may be associated with the clinical remission rate at week 14 in the univariate analysis, the multivariate analysis revealed no significant associations between these factors. Other factors, such as age, sex, BMI, disease duration and location, Mayo Clinic score, extraintestinal manifestations, prior medication use, and opportunistic infection, were not significantly associated with the primary and secondary outcomes (Tables 3, 4; Supplementary Tables S1–S3).

## Discussion

To the best of our knowledge, this is the first study to evaluate the efficacy and safety of CS combined with IFX as first-line therapy for patients with ASUC with mucosal deficiency. Our findings suggest that, when compared to IFX as a sequential rescue therapy after CS, CS combined with IFX as an initial treatment may represent a better therapeutic option for patients with ASUC with mucosal deficiency, who are at a higher risk of adverse treatment outcomes.

For decades, intravenous CS have been recommended as the initial standard treatment for patients with ASUC; however, approximately one-third of patients do not respond adequately (5). More recently, IFX has been proven to be the preferred rescue therapy because it can be used as maintenance therapy and has a superior adverse event profile. However, according to data from previous randomized clinical trials (RCTs), the short-term response rate of steroid-refractory ASUC to IFX ranged from 46 to 83%, and the colectomy rate ranged from 0 to 50% (3). Based on the available data, various rescue strategies involving intensified IFX dosing have been explored, including initiating doses of 10 mg/kg or accelerated induction IFX therapy (12–14). However, the efficacy of shorter dosing intervals and higher doses of IFX remains uncertain (1, 15, 16), and there still exists a significant rate of treatment failure leading to colectomy. This highlights the need for more effective therapies for patients admitted with ASUC.

In our study, we focused on a special type of ASUC, known as ASUC with mucosal deficiency. In this form of ASUC, the colonic mucosa layer is absent, and the muscular layer is exposed on colonoscopy. Patients with ASUC with mucosal deficiency represent a more challenging clinical scenario due to the higher risk of requiring urgent surgical intervention for uncontrolled disease. Therefore, a more effective initial treatment strategy is crucial for these patients. Up until now, most previous studies have focused on rescue therapy for ASUC, including small-molecule Janus kinase (JAK) inhibitors such as tofacitinib and upadacitinib, as well as other biologics such as vedolizumab and ustekinumab (3, 8, 10, 17). Few studies have focused on new first-line strategies for ASUC. Given this, we conducted this study to evaluate the efficacy of intravenous CS combined with IFX as a first-line treatment for patients with ASUC with mucosal deficiency.

In this study, we found that compared to the CS sequential IFX strategy, the initial CS combined with the IFX regimen had higher rates of endoscopic improvement at week 14 (84.0% vs. 50.0%) and durable clinical remission (85.7% vs. 35.7%) in the patients with ASUC with mucosa deficiency. Furthermore, the multivariate analysis demonstrated that the CS combined with the IFX regimen was well established as an independent predictor of a higher endoscopic improvement rate at week 14 (OR = 8.428) and a durable clinical remission rate (OR = 10.800).

This finding has important implications, as patients with ASUC with mucosal deficiency are more likely to be advised to undergo colectomy based on their admission and endoscopic characteristics. Endoscopic remission in the short term may reduce the risk of colectomy for these patients. Although the difference in the colectomy rates within 90 days was not statistically significant between the two strategies in this study, the smaller sample sizes in each group might have reduced the statistical power. Numerically, the colectomy rate within 90 days was lower in the combined group than in the sequential group (4.0% vs. 11.1%). The higher durable clinical remission rate further confirmed a good long-term prognosis in the combined group.

Other potent risk factors related to clinical and endoscopic efficacy were also explored in our study. Finally, a serum albumin level greater than 25 g/L at induction was established as an independent predictor of higher rates of clinical remission (OR = 10.625) and endoscopic improvement at week 14 (OR = 7.685). These results are consistent with findings from previous studies (18, 19).

While this study was not powered to evaluate the safety, we did not observe any increased risk of infection or other adverse events in the patients treated with the initial CS combined with the IFX regimen.

Our study has some limitations. First, it was a non-randomized, retrospective study with a relatively small size in our ASUC with mucosal deficiency cohort. It reduced our ability to identify small differences in efficacy and safety. Second, the data for the study were derived from a single large tertiary IBD center, which may have limited the generalizability of our study findings to other centers.

Despite these limitations, this study suggests that initial CS combined with IFX may be an effective therapeutic strategy for patients with ASUC with mucosal deficiency. It also suggests that better outcomes may be achieved when CS combined with IFX is administered as first-line therapy rather than using IFX as salvage therapy. This study is one of the few studies exploring first-line therapy for ASUC and provides the current best evidence for the use of initial CS combined with IFX in patients with ASUC with mucosal deficiency. Another recent study also focused on adding tofacitinib to CS in patients with ASUC. They found that the combination of tofacitinib and CS improved treatment responsiveness and reduced the need for IFX rescue therapy on day 7 (20).

In conclusion, CS combined with IFX as first-line therapy may be an effective and safe induction strategy for patients with ASUC with mucosal deficiency. Larger, prospective, multicenter RCTs are needed to further evaluate the efficacy and safety of this strategy for patients with ASUC.

## Data Availability

The original contributions presented in the study are included in the article/Supplementary material, further inquiries can be directed to the corresponding authors.
