# Supplementary material for: Corticosteroids combined with infliximab vs. corticosteroids sequential infliximab for acute severe ulcerative colitis with mucosal deficiency: a retrospective study
Source: Front Med (Lausanne). 2024 Nov 20;11:1442519. doi: 10.3389/fmed.2024.1442519 (PMC11614599; doi:10.3389/fmed.2024.1442519)
Supplement: Supplementary file 1 [file Table_1.DOCX]

**Supplementary Table 1 Univariate and multivariate analyses of the risk factor of 14w clinical remission in subjects according to the analyzed variables**

|  | **14w clinical remission** |  | |  | | **Univariate analysis** | | | | | | |  | | **Multivariate analysis** | | | | |
| --- | --- | --- | --- | --- | --- | --- | --- | --- | --- | --- | --- | --- | --- | --- | --- | --- | --- | --- | --- |
|  | **n=34** | |  | | **OR** | | **95% CI** | | ***P* value** | |  | | | **OR** | | **95% CI** | | ***P* value** | |
| Age (y), n (%) | | | | | | | | | | | | | | | | | | | |
| ≤40 (n=19) | 18 (94.7) |  | | 1 | | | |  | |  | |  |  | | | |  | |  |
| >40 (n=24) | 16 (66.7) |  | | 0.111 | | | | 0.012-0.988 | | 0.049 | |  | 0.961 | | | | 0.893-1.033 | | 0.280 |
| Gender, n (%) | | | | | | | | | | | | | | | | | | | |
| Male (n=27) | 21 (77.8) |  | | 1 | | | |  | |  | |  |  | | | |  | |  |
| Female (n=16) | 13 (81.3) |  | | 1.238 | | | | 0.263-5.827 | | 0.787 | |  |  | | | |  | |  |
| BMI (Kg/m^2^), n (%) | | | | | | | | | | | | | | | | | | | |
| ≤20 (n=16) | 12 (75.0) |  | | 1 | | | |  | |  | |  |  | | | |  | |  |
| >20 (n=27) | 22 (81.5) |  | | 1.467 | | | | 0.330-6.515 | | 0.615 | |  |  | | | |  | |  |
| Disease duration (y), n (%) | | | | | | | | | | | | | | | | | | | |
| <1 (n=19) | 16 (84.2) |  | | 1 | | | |  | |  | |  |  | | | |  | |  |
| ≥1 (n=24) | 18 (75.0) |  | | 0.563 | | | | 0.120-2.626 | | 0.464 | |  |  | | | |  | |  |
| Disease location, n (%) | | | | | | | | | | | | | | | | | | | |
| E2 (n=4) | 2 (50.0) |  | | 1 | | | |  | |  | |  |  | | | |  | |  |
| E3 (n=39) | 32 (82.1) |  | | 4.571 | | | | 0.547-38.228 | | 0.161 | |  |  | | | |  | |  |
| Mayo Clinic score, n (%) | | | | | | | | | | | | | | | | | | | |
| 11 (n=35) | 28 (80.0) |  | | 1 | | | |  | |  | |  |  | | | |  | |  |
| 12 (n=8) | 6 (75.0) |  | | 0.750 | | | | 0.124-4.546 | | 0.754 | |  |  | | | |  | |  |
| Extraintestinal manifestations, n (%) | | | | | | | | | | | | | | | | | | | |
| No (n=34) | 27 (79.4) |  | | 1 | | | |  | |  | |  |  | | | |  | |  |
| Yes (n=9) | 7 (77.8) |  | | 0.907 | | | | 0.153-5.369 | | 0.915 | |  |  | | | |  | |  |
| Prior oral mesalamine use, n (%) | | | | | | | | | | | | | | | | | | | |
| No (n=1) | 1 (100.0) |  | | 1 | | | |  | |  | |  |  | | | |  | |  |
| Yes (n=42) | 33 (78.6) |  | | 0.000 | | | | - | | 1.000 | |  |  | | | |  | |  |
| Prior systematic steroids use, n (%) | | | | | | | | | | | | | | | | | | | |
| No (n=16) | 13 (81.3) |  | | 1 | | | |  | |  | |  |  | | | |  | |  |
| Yes (n=27) | 21 (77.8) |  | | 0.808 | | | | 0.172-3.801 | | 0.787 | |  |  | | | |  | |  |
| Prior azathioprine use, n (%) | | | | | | | | | | | | | | | | | | | |
| No (n=39) | 31 (79.5) |  | | 1 | | | |  | |  | |  |  | | | |  | |  |
| Yes (n=4) | 3 (75.0) |  | | 0.774 | | | | 0.071-8.474 | | 0.834 | |  |  | | | |  | |  |
| Prior biologics use, n (%) | | | | | | | | | | | | | | | | | | | |
| No (n=34) | 28 (82.4) |  | | 1 | | | |  | |  | |  |  | | | |  | |  |
| Yes (n=9) | 6 (66.7) |  | | 0.429 | | | | 0.083-2.215 | | 0.312 | |  |  | | | |  | |  |
| Fever, n (%) | | | | | | | | | | | | | | | | | | | |
| No (n=19) | 12 (63.2) |  | | 1 | | | |  | |  | |  |  | | | |  | |  |
| Yes (n=24) | 22 (91.7) |  | | 6.417 | | | | 1.147-35.895 | | 0.034 | |  | 5.531 | | | | 0.655-46.673 | | 0.116 |
| Abdominal tenderness, n (%) | | | | | | | | | | | | | | | | | | | |
| No (n=41) | 32 (78.0) |  | | 1 | | | |  | |  | |  |  | | | |  | |  |
| Yes (n=2) | 2 (100.0) |  | | - | | | | - | | 0.999 | |  |  | | | |  | |  |
| CRP at induction (mg/L), n (%) | | | | | | | | | | | | | | | | | | | |
| ≤40 (n=18) | 12 (66.7) |  | | 1 | | | |  | |  | |  |  | | | |  | |  |
| >40 (n=25) | 22 (88.0) |  | | 3.667 | | | | 0.775-17.348 | | 0.101 | |  | 4.424 | | | | 0.599-32.645 | | 0.145 |
| Albumin at induction (g/L), n (%) | | | | | | | | | | | | | | | | | | | |
| ≤25 (n=9) | 5 (55.6) |  | | 1 | | | |  | |  | |  |  | | | |  | |  |
| >25 (n=34) | 29 (85.3) |  | | 4.640 | | | | 0.917-23.483 | | 0.064 | |  | 10.625 | | | | 1.258-89.737 | | 0.030 |
| CRP/albumin ratio, n (%) | | | | | | | | | | | | | | | | | | | |
| ≤1.7 (n=22) | 16 (72.7) |  | | 1 | | | |  | |  | |  |  | | | |  | |  |
| >1.7 (n=21) | 18 (85.7) |  | | 2.250 | | | | 0.482-10.504 | | 0.302 | |  |  | | | |  | |  |
| C. difficile infection, n (%) | | | | | | | | | | | | | | | | | | | |
| No (n=31) | 26 (83.9) |  | | 1 | | | |  | |  | |  |  | | | |  | |  |
| Yes (n=12) | 8 (66.7) |  | | 0.385 | | | | 0.083-1.785 | | 0.222 | |  |  | | | |  | |  |
| CMV infection n (%) | | | | | | | | | | | | | | | | | | | |
| No (n=22) | 18 (81.8) |  | | 1 | | | |  | |  | |  |  | | | |  | |  |
| Yes (n=21) | 16 (76.2) |  | | 0.711 | | | | 0.162-3.115 | | 0.651 | |  |  | | | |  | |  |
| EBV infection, n (%) | | | | | | | | | | | | | | | | | | | |
| No (n=36) | 30 (83.3) |  | | 1 | | | |  | |  | |  |  | | | |  | |  |
| Yes (n=7) | 4 (57.1) |  | | 0.267 | | | | 0.047-1.511 | | 0.135 | |  |  | | | |  | |  |
| Therapy, n (%) | | | | | | | | | | | | | | | | | | | |
| CS sequential IFX (n=18) | 13 (72.2) |  | | 1 | | | |  | |  | |  |  | | | |  | |  |
| CS combined IFX (n=25) | 21 (84.0) |  | | 2.019 | | | | 0.457-8.920 | | 0.354 | |  |  | | | |  | |  |

BMI, Body Mass Index; E2, Left-sided colitis; E3, Extensive colitis; CRP, C-reactive protein; CMV, cytomegalovirus; EBV, Epstein-Barr Virus; CS, corticosteroids; IFX, infliximab.

**Supplementary Table 2 Univariate and multivariate analyses of the risk factor of 14w endoscopic remission in subjects according to the analyzed variables**

|  | **14w endoscopic remission** |  |  | | **Univariate analysis** | | | | | | |  | | **Multivariate analysis** | | | | | |
| --- | --- | --- | --- | --- | --- | --- | --- | --- | --- | --- | --- | --- | --- | --- | --- | --- | --- | --- | --- |
|  | **n=10** |  | | **OR** | | **95% CI** | | ***P* value** | |  | | | **OR** | | **95% CI** | | ***P* value** | |  |
| Age (y), n (%) | | | | | | | | | | | | | | | | | | | |
| ≤40 (n=19) | 3 (15.8) |  | 1 | | | |  | |  | |  |  | | | |  | |  | |
| >40 (n=24) | 7 (29.2) |  | 2.196 | | | | 0.483-9.991 | | 0.309 | |  |  | | | |  | |  | |
| Gender, n (%) | | | | | | | | | | | | | | | | | | | |
| Male (n=27) | 5 (18.5) |  | 1 | | | |  | |  | |  |  | | | |  | |  | |
| Female (n=16) | 5 (31.3) |  | 2.000 | | | | 0.476-8.403 | | 0.344 | |  |  | | | |  | |  | |
| BMI (Kg/m^2^), n (%) | | | | | | | | | | | | | | | | | | | |
| ≤20 (n=16) | 4 (25.0) |  | 1 | | | |  | |  | |  |  | | | |  | |  | |
| >20 (n=27) | 6 (22.2) |  | 0.857 | | | | 0.201-3.656 | | 0.835 | |  |  | | | |  | |  | |
| Disease duration (y), n (%) | | | | | | | | | | | | | | | | | | | |
| <1 (n=19) | 7 (36.8) |  | 1 | | | |  | |  | |  |  | | | |  | |  | |
| ≥1 (n=24) | 3 (12.5) |  | 0.245 | | | | 0.053-1.128 | | 0.071 | |  | 0.245 | | | | 0.053-1.128 | | 0.071 | |
| Disease location, n (%) | | | | | | | | | | | | | | | | | | | |
| E2 (n=4) | 0 (0.0) |  | 1 | | | |  | |  | |  |  | | | |  | |  | |
| E3 (n=39) | 10 (25.6) |  | - | | | | - | | 0.999 | |  |  | | | |  | |  | |
| Mayo Clinic score, n (%) | | | | | | | | | | | | | | | | | | | |
| 11 (n=35) | 10 (28.6) |  | 1 | | | |  | |  | |  |  | | | |  | |  | |
| 12 (n=8) | 0 (0.0) |  | - | | | | - | | 0.999 | |  |  | | | |  | |  | |
| Extraintestinal manifestations, n (%) | | | | | | | | | | | | | | | | | | | |
| No (n=34) | 10 (29.4) |  | 1 | | | |  | |  | |  |  | | | |  | |  | |
| Yes (n=9) | 0 (0.0) |  | - | | | | - | | 0.999 | |  |  | | | |  | |  | |
| Prior oral mesalamine use, n (%) | | | | | | | | | | | | | | | | | | | |
| No (n=1) | 1 (100.0) |  | 1 | | | |  | |  | |  |  | | | |  | |  | |
| Yes (n=42) | 9 (21.4) |  | 0.000 | | | | - | | 1.000 | |  |  | | | |  | |  | |
| Prior systematic steroids use, n (%) | | | | | | | | | | | | | | | | | | | |
| No (n=16) | 2 (12.5) |  | 1 | | | |  | |  | |  |  | | | |  | |  | |
| Yes (n=27) | 8 (29.6) |  | 2.947 | | | | 0.540-16.074 | | 0.212 | |  |  | | | |  | |  | |
| Prior azathioprine use, n (%) | | | | | | | | | | | | | | | | | | | |
| No (n=39) | 9 (23.1) |  | 1 | | | |  | |  | |  |  | | | |  | |  | |
| Yes (n=4) | 1 (25.0) |  | 1.111 | | | | 0.103-12.037 | | 0.931 | |  |  | | | |  | |  | |
| Prior biologics use, n (%) | | | | | | | | | | | | | | | | | | | |
| No (n=34) | 9 (26.5) |  | 1 | | | |  | |  | |  |  | | | |  | |  | |
| Yes (n=9) | 1 (11.1) |  | 0.347 | | | | 0.038-3.178 | | 0.349 | |  |  | | | |  | |  | |
| Fever, n (%) | | | | | | | | | | | | | | | | | | | |
| No (n=19) | 3 (15.8) |  | 1 | | | |  | |  | |  |  | | | |  | |  | |
| Yes (n=24) | 7 (29.2) |  | 2.196 | | | | 0.483-9.991 | | 0.309 | |  |  | | | |  | |  | |
| Abdominal tenderness, n (%) | | | | | | | | | | | | | | | | | | | |
| No (n=41) | 9 (22.0) |  | 1 | | | |  | |  | |  |  | | | |  | |  | |
| Yes (n=2) | 1 (50.0) |  | 3.556 | | | | 0.202-62.632 | | 0.386 | |  |  | | | |  | |  | |
| CRP at induction (mg/L), n (%) | | | | | | | | | | | | | | | | | | | |
| ≤40 (n=18) | 3 (16.7) |  | 1 | | | |  | |  | |  |  | | | |  | |  | |
| >40 (n=25) | 7 (28.0) |  | 1.944 | | | | 0.427-8.856 | | 0.390 | |  |  | | | |  | |  | |
| Albumin at induction (g/L), n (%) | | | | | | | | | | | | | | | | | | | |
| ≤25 (n=9) | 2 (22.2) |  | 1 | | | |  | |  | |  |  | | | |  | |  | |
| >25 (n=34) | 8 (23.5) |  | 1.077 | | | | 0.185-6.259 | | 0.934 | |  |  | | | |  | |  | |
| CRP/albumin ratio, n (%) | | | | | | | | | | | | | | | | | | | |
| ≤1.7 (n=22) | 6 (27.3) |  | 1 | | | |  | |  | |  |  | | | |  | |  | |
| >1.7 (n=21) | 4 (19.0) |  | 0.627 | | | | 0.149-2.642 | | 0.525 | |  |  | | | |  | |  | |
| C. difficile infection, n (%) | | | | | | | | | | | | | | | | | | | |
| No (n=31) | 8 (25.8) |  | 1 | | | |  | |  | |  |  | | | |  | |  | |
| Yes (n=12) | 2 (16.7) |  | 0.575 | | | | 0.103-3.205 | | 0.528 | |  |  | | | |  | |  | |
| CMV infection n (%) | | | | | | | | | | | | | | | | | | | |
| No (n=22) | 5 (22.7) |  | 1 | | | |  | |  | |  |  | | | |  | |  | |
| Yes (n=21) | 5 (23.8) |  | 1.062 | | | | 0.258-4.374 | | 0.933 | |  |  | | | |  | |  | |
| EBV infection, n (%) | | | | | | | | | | | | | | | | | | | |
| No (n=36) | 9 (25.0) |  | 1 | | | |  | |  | |  |  | | | |  | |  | |
| Yes (n=7) | 1 (14.3) |  | 0.500 | | | | 0.053-4.732 | | 0.546 | |  |  | | | |  | |  | |
| Therapy, n (%) | | | | | | | | | | | | | | | | | | | |
| CS sequential IFX (n=18) | 2 (11.1) |  | 1 | | | |  | |  | |  |  | | | |  | |  | |
| CS combined IFX (n=25) | 8 (32.0) |  | 3.765 | | | | 0.692-20.468 | | 0.125 | |  |  | | | |  | |  | |

BMI, Body Mass Index; E2, Left-sided colitis; E3, Extensive colitis; CRP, C-reactive protein; CMV, cytomegalovirus; EBV, Epstein-Barr Virus; CS, corticosteroids; IFX, infliximab.

**Supplementary Table 3 Univariate and multivariate analyses of the risk factor of 90d colectomy in subjects according to the analyzed variables**

|  | **90d colectomy** |  |  | | **Univariate analysis** | | | | | | |  | | **Multivariate analysis** | | | | | |
| --- | --- | --- | --- | --- | --- | --- | --- | --- | --- | --- | --- | --- | --- | --- | --- | --- | --- | --- | --- |
|  | **n=3** |  | | **OR** | | **95% CI** | | ***P* value** | |  | | | **OR** | | **95% CI** | | ***P* value** | |  |
| Age (y), n (%) | | | | | | | | | | | | | | | | | | | |
| ≤40 (n=19) | 0 (0.0) |  | 1 | | | |  | |  | |  |  | | | |  | |  | |
| >40 (n=24) | 3 (12.5) |  | - | | | | - | | 0.998 | |  |  | | | |  | |  | |
| Gender, n (%) | | | | | | | | | | | | | | | | | | | |
| Male (n=27) | 1 (3.7) |  | 1 | | | |  | |  | |  |  | | | |  | |  | |
| Female (n=16) | 2 (12.5) |  | 3.714 | | | | 0.309-44.657 | | 0.301 | |  |  | | | |  | |  | |
| BMI (Kg/m^2^), n (%) | | | | | | | | | | | | | | | | | | | |
| ≤20 (n=16) | 1 (6.3) |  | 1 | | | |  | |  | |  |  | | | |  | |  | |
| >20 (n=27) | 2 (7.4) |  | 1.200 | | | | 0.100-14.392 | | 0.886 | |  |  | | | |  | |  | |
| Disease duration (y), n (%) | | | | | | | | | | | | | | | | | | | |
| <1 (n=19) | 2 (10.5) |  | 1 | | | |  | |  | |  |  | | | |  | |  | |
| ≥1 (n=24) | 1 (4.2) |  | 0.370 | | | | 0.031-4.417 | | 0.432 | |  |  | | | |  | |  | |
| Disease location, n (%) | | | | | | | | | | | | | | | | | | | |
| E2 (n=4) | 0 (0.0) |  | 1 | | | |  | |  | |  |  | | | |  | |  | |
| E3 (n=39) | 3 (7.7) |  | - | | | | - | | 0.999 | |  |  | | | |  | |  | |
| Mayo Clinic score, n (%) | | | | | | | | | | | | | | | | | | | |
| 11 (n=35) | 3 (8.6) |  | 1 | | | |  | |  | |  |  | | | |  | |  | |
| 12 (n=8) | 0 (0.0) |  | - | | | | - | | 0.999 | |  |  | | | |  | |  | |
| Extraintestinal manifestations, n (%) | | | | | | | | | | | | | | | | | | | |
| No (n=34) | 1 (2.9) |  | 1 | | | |  | |  | |  |  | | | |  | |  | |
| Yes (n=9) | 2 (22.2) |  | 9.429 | | | | 0.747-118.982 | | 0.083 | |  | - | | | | - | | 0.998 | |
| Prior oral mesalamine use, n (%) | | | | | | | | | | | | | | | | | | | |
| No (n=1) | 0 (0.0) |  | 1 | | | |  | |  | |  |  | | | |  | |  | |
| Yes (n=42) | 3 (7.1) |  | - | | | | - | | 1.000 | |  |  | | | |  | |  | |
| Prior systematic steroids use, n (%) | | | | | | | | | | | | | | | | | | | |
| No (n=16) | 1 (6.3) |  | 1 | | | |  | |  | |  |  | | | |  | |  | |
| Yes (n=27) | 2 (7.4) |  | 1.200 | | | | 0.100-14.392 | | 0.886 | |  |  | | | |  | |  | |
| Prior azathioprine use, n (%) | | | | | | | | | | | | | | | | | | | |
| No (n=39) | 3 (7.7) |  | 1 | | | |  | |  | |  |  | | | |  | |  | |
| Yes (n=4) | 0 (0.0) |  | - | | | | - | | 0.999 | |  |  | | | |  | |  | |
| Prior biologics use, n (%) | | | | | | | | | | | | | | | | | | | |
| No (n=34) | 2 (5.9) |  | 1 | | | |  | |  | |  |  | | | |  | |  | |
| Yes (n=9) | 1 (11.1) |  | 2.000 | | | | 0.161-24.916 | | 0.590 | |  |  | | | |  | |  | |
| Fever, n (%) | | | | | | | | | | | | | | | | | | | |
| No (n=19) | 2 (10.5) |  | 1 | | | |  | |  | |  |  | | | |  | |  | |
| Yes (n=24) | 1 (4.2) |  | 0.370 | | | | 0.031-4.417 | | 0.432 | |  |  | | | |  | |  | |
| Abdominal tenderness, n (%) | | | | | | | | | | | | | | | | | | | |
| No (n=41) | 3 (7.3) |  | 1 | | | |  | |  | |  |  | | | |  | |  | |
| Yes (n=2) | 0 (0.0) |  | - | | | | - | | 0.999 | |  |  | | | |  | |  | |
| CRP at induction (mg/L), n (%) | | | | | | | | | | | | | | | | | | | |
| ≤40 (n=18) | 2 (11.1) |  | 1 | | | |  | |  | |  |  | | | |  | |  | |
| >40 (n=25) | 1 (4.0) |  | 0.333 | | | | 0.028-3.990 | | 0.386 | |  |  | | | |  | |  | |
| Albumin at induction (g/L), n (%) | | | | | | | | | | | | | | | | | | | |
| ≤25 (n=9) | 2 (44.4) |  | 1 | | | |  | |  | |  |  | | | |  | |  | |
| >25 (n=34) | 1 (76.5) |  | 0.106 | | | | 0.008-1.338 | | 0.083 | |  | - | | | | - | | 0.998 | |
| CRP/albumin ratio, n (%) | | | | | | | | | | | | | | | | | | | |
| ≤1.7 (n=22) | 2 (9.1) |  | 1 | | | |  | |  | |  |  | | | |  | |  | |
| >1.7 (n=21) | 1 (4.8) |  | 0.500 | | | | 0.042-5.966 | | 0.584 | |  |  | | | |  | |  | |
| C. difficile infection, n (%) | | | | | | | | | | | | | | | | | | | |
| No (n=31) | 2 (6.5) |  | 1 | | | |  | |  | |  |  | | | |  | |  | |
| Yes (n=12) | 1 (8.3) |  | 1.318 | | | | 0.108-16.039 | | 0.828 | |  |  | | | |  | |  | |
| CMV infection n (%) | | | | | | | | | | | | | | | | | | | |
| No (n=22) | 2 (9.1) |  | 1 | | | |  | |  | |  |  | | | |  | |  | |
| Yes (n=21) | 1 (4.8) |  | 0.500 | | | | 0.042-5.966 | | 0.584 | |  |  | | | |  | |  | |
| EBV infection, n (%) | | | | | | | | | | | | | | | | | | | |
| No (n=36) | 2 (5.6) |  | 1 | | | |  | |  | |  |  | | | |  | |  | |
| Yes (n=7) | 1 (14.3) |  | 2.833 | | | | 0.221-36.379 | | 0.424 | |  |  | | | |  | |  | |
| Therapy, n (%) | | | | | | | | | | | | | | | | | | | |
| CS sequential IFX (n=18) | 2 (11.1) |  | 1 | | | |  | |  | |  |  | | | |  | |  | |
| CS combined IFX (n=25) | 1 (4.0) |  | 0.333 | | | | 0.028-3.990 | | 0.386 | |  |  | | | |  | |  | |

BMI, Body Mass Index; E2, Left-sided colitis; E3, Extensive colitis; CRP, C-reactive protein; CMV, cytomegalovirus; EBV, Epstein-Barr Virus; CS, corticosteroids; IFX, infliximab.
